# Thermal analysis of Al + 0.1% CNT ribbon

**DOI:** 10.1186/s11671-015-0878-3

**Published:** 2015-04-08

**Authors:** Sergiy Revo, Smail Hamamda, Kateryna Ivanenko, Oleh Boshko, Ahmed Djarri, Abdelhamid Boubertakh

**Affiliations:** Taras Shevchenko National University of Kyiv, 64/13, Volodymyrska Street, 01601 Kyiv, Ukraine; Laboratory of Thermodynamics and Surface Treatment of Materials, Constantine University 1, B.P. 325 Route Ain El Bey, Constantine, 25017 Algeria

**Keywords:** Nanomaterial, Ribbon, Nanotube, Carbon, Thermal expansion, Anisotropy

## Abstract

The objective of this work is a dilatometric study of Al + 0.1% of multiwall carbon nanotubes nanocomposite material (NCM) in three directions: X - parallel to the rolling direction; Y - perpendicular to the rolling direction and (Z) perpendicular to the ribbon plane. NCM specimens were made in the form of a 0.1-mm-thick ribbon. The temperature range used for measurements was 20°C to 600°C. The obtained results show that presence of nanotubes affects the thermal expansion coefficient (TEC) measured in different directions. α_x_(T) and α_y_(T) - TEC plots as a function of temperature along X and Y directions, respectively - have substantially the same shape and overlap in the area of 400°C. The expansion along X-axis becomes greater than along Y-axis below this temperature value. It is clear that the coefficient α_z_(T) is lower than α_x_(T) and α_y_(T) over the entire temperature range. The expansion along Z-axis is smaller compared to that along X- and Y-axes. This behaviour suggests that there is a strong interatomic interaction along this direction (Z). α_z_(T) becomes monotonous and constant and is equal to 8 × 10^−6^°C^−1^ at temperatures above 300°C. Such order of magnitude had not been obtained in earlier studies of aluminium alloys. The obtained TEC shows high anisotropy, which grows with the increase of temperature. The heat flow (differential scanning calorimetry, (DSC)) of Al + 0.1% carbon nanotubes (CNT) NCM is more intense compared to that of pure aluminium produced in similar conditions. The two representative curves have similar shape and are almost entirely overlapped. The thermogravimetry results confirm those of DSC. The Raman spectrum of this nanomaterial shows that intensity of G and D bonds is significantly increased compared to that of the pure material. The infrared diagram also confirms that in this case the mentioned bonds are more intensive NCM. The tensile strength measurements (σB) of the studied NCM also demonstrate that its value increases from 140 ± 10 MPa for Al without nanotubes to 200 ± 10 MPa for NCM.

## Background

In the recent years, the research in material science became faster owing to development of new technical methods for production of alloys and nanocomposite materials (NCM) [1-3]. Many expensive materials can now be replaced with composites containing different types of carbon nanotubes. They have become essential in several strategic sectors of the economy due to significant improvements in their physical properties and mechanical properties in particular. Despite a quantitative and qualitative breakthrough in the use of this new class of materials, aluminium has not lost its key role in the materials science [4]. Its use is still essential in many fields of industry, especially in transportation, aircraft building and space.

In 1980s, a rapid crystallization method was used to produce aluminium alloys with microstructure, which provided for high mechanical properties compared to conventional aluminium alloys.

Employment of quick-chilling methods, such as direct chill casting (DCC) and melt spinning (single, twin roller), was useful for the development of new methods for production of aluminium alloys with quite interesting thermodynamic properties. Their thermal expansion coefficient α(T) was lower compared to that of pure aluminium and conventional aluminium alloys [5]. The thermal expansion coefficients of specimens of alloys, made using DCC method along the rolling direction and perpendicular to the rolling plane direction, was lower compared to pure aluminium and conventional alloys. The same studies and procedures were applied to aluminium alloys in the form of sheets and ribbons. Dilatometric measurements have proved a decrease of the thermal expansion coefficient both for sheets and ribbons of aluminium alloys produced by casting. α(T) has become quite isotropic magnitude.

The invention of carbon nanotubes (CNT) and the method of CNT incorporation in aluminium have opened up many opportunities for direct use in everyday life. Incorporation of nanotubes in aluminium is the origin of some interesting physical and primarily physico-mechanical properties. Several studied have proved an improvement of other properties and a positive role by CNT in the metallic matrix [6].

Some authors have shown in their works that CNT are effective and promising for employment in metallomatrix composites. Thus, in particular, adding CNT to aluminium increases the coefficient of damping, with no loss in the strength or rigidity of the metallic matrix [7]. In reference [8], the authors have proved a positive role of adding a small amount of multiwall CNT and attributed the improvements to high dispersion of CNT. In reference [9], the authors show that adding CNT to the aluminium matrix enhances the mechanical properties, the yield strength, the tensile strength and the microhardness, while they also state a 10% decrement of the thermal expansion coefficient of this material.

Analysis of data from literature sources by different authors shows that the thermal expansion coefficient as a function of temperature for composites containing CNT is decreased in comparison with this value for the pure matrix. All these measurements were made in a unique direction. The objective of this study was to find the thermal expansion coefficient as a function of temperature, measured in different directions of the nanocomposite material based on aluminium, which contains multiwall carbon nanotubes. The studied nanocomposite is a ribbon.

## Methods

### Specimen preparation

A999 aluminium powder and multiwall CNT were used as components of Al-CNT nanocomposite material. CNT specimens were obtained by pressing and sintering of a mixture of the components in the argon atmosphere at the temperature *Т* = 380°C ± 5°C during 10 min and subsequent rolling of the produced piece. The optimal CNT concentration in the mixture was of the order of 0.1 vol.%. Sintering of the specimens was done in a mould at the pressure of 45 MPa, and rolling was produced at room temperature in such a way that relative deformation after the first passing through the rollers was of the order of 20% to 30%.

The multiwall carbon nanotubes were produced by CVD method in a rotating reactor [10]. The average size of carbon nanotubes is 10 to 20 nm, and the specific surface area, which was calculated by desorption of argon, was 200 to 400 m^2^/g, while their bulk density was from 20 to 40 g/dm^3^.

### Equipment used

– Netzsch 402C dilatometer (NETZSCH, Selb, Germany) with the accuracy of 3% was used in this study. We put together two Al + 0.1% CNT ribbon pieces for measurements of α(T) along X- and Y-axes and took one piece of ribbon in the Z-axis direction (perpendicular to the rolling plane). The heating rate used was 10°C/min. The thermal expansion coefficient was measured in the temperature range of 25°C to 600°C.

– Jupiter STA 449 F3 calorimeter by Netzsch (NETZSCH, Selb, Germany) was used for thermal analysis with the use of differential scanning calorimetry (DSC) and thermogravimetry (TG). The same heating rate as in the dilatometric measurements was used.

– The devices used to obtain spectra were Jasco FT/IR-6300 (Jasco Analytical Instruments, Easton, MD, USA) for the infrared spectra and Bruker SENTERRA (Bruker, Billerica, MA, USA) for the Raman ones.

## Results and discussion

**Figure 1:**
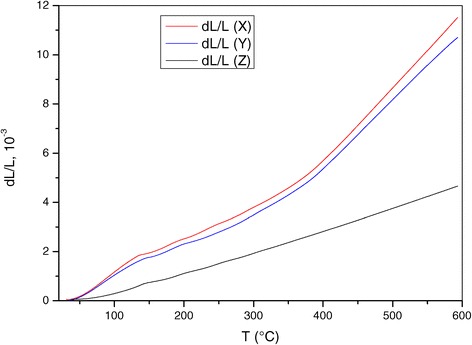
**Elongation relative change of nanocomposite Al + 0.1% CNT along X, Y and Z directions.**

Figure 1 presents relative elongation changes of specimens ΔL/L as a function of temperature measured along the three directions (X, Y and Z). The dilatometric behaviour of NCM varies from one direction to another. The ΔL/L curves in X- and Y-directions present the same shape but their intensity changes. The intensity of ΔL/L variation along Z-direction is significantly smaller than along X- and Y-axes over the whole temperature range.

**Figure 2:**
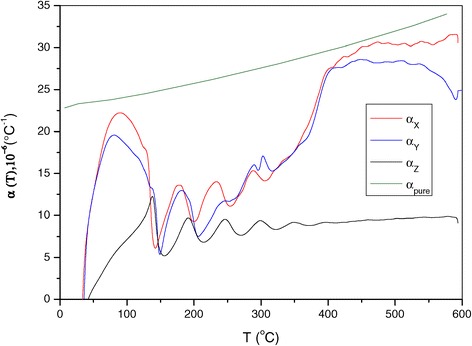
**Thermal expansion coefficient of Al + 0.1% CNT and pure aluminium along the three directions X, Y and Z.**

The thermal expansion coefficients α_x_(T), α_y_(T) and α_z_(T) show that the three dilatometric curves (Figure 2) are different over the entire temperature range.

The curves of α_x_(T) and α_y_(T) reach their dilatometric peaks at approximately 90°C with intensity which varies depending on the direction of the measurement. It is clear that α_x_(T) is higher than α_y_(T). The two curves overlap from 110°C to 360°C, and the average TEC is equal to 15 × 10^−6^°C^−1^. α_x_(T) becomes greater than α_y_(T) above 360°C and over the rest of the temperature range. Starting from the temperature of 600°C, α_y_(T) equals 25 × 10^−6^°C^−1^, while α_x_(T) reaches the value of 30 × 10^−6^°C^−1^. α_z_(T) curve does not contain the dilatometric anomalies observed in the previous two directions.

α_z_(T) is smaller than α_x_(T) and α_y_(T) over the entire temperature range. After the temperature of 340°C, TEC along the Z-axis becomes monotonous, varies linearly up to 600°C and does not exceed 8 × 10^−6^°C^−1^. The obtained value of the thermal expansion coefficient has been achieved for the first time. This value is at least three times smaller than TEC of conventional aluminium alloys and two to three times smaller than TEC of similar aluminium materials produced by quick-chilling methods. We believe that a significant decrement of the thermal expansion coefficient over a wide temperature range and presence of a section where α_z_(T) is constant represent an original, interesting and promising result.

In order to emphasize the results of this work, we have done a comparison with other research works in this area. In work [11], the authors studied the effect of introducing CNT into 2009Al matrix. They show that the yield stress has improved for the studied material, and the thermal expansion coefficient of the nanocomposite has decreased. Paper [12] shows that nanotubes in Ni matrix modify its mechanical and thermal properties. Expansion of the composite reinforced with multiwall carbon nanotubes has seen a 12% drop compared to pure Ni. In the same manner, in reference [13] was shown the decreasing at least of 12% of the expansion of 2024Al matrix, wherein multiwall carbon nanotubes have been introduced.

The results of our study, as well as the studies by other authors, are unanimous in proving a decrease in the thermal expansion coefficient of aluminium containing nanotubes. The literature sources on dilatometric studies of aluminium with nanotubes describe TEC measurements taken only in one unique direction, whereas our studies underline the importance of the directions used for measuring. We have taken into account the anisotropy effect.

**Figure 3:**
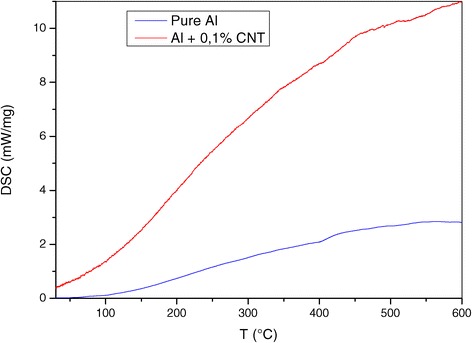
**Differential scanning calorimetry of nanocomposite Al + 0.1% CNT and pure aluminium.**

**Figure 4:**
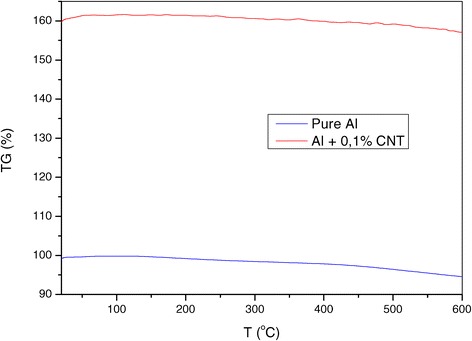
**Thermogravimetry of nanocomposite Al + 0.1% CNT and pure aluminium.**

Another interesting finding of our study is a lack of anomaly on dilatometric curves of α_x_(T), α_y_(T) and α_z_(T) at high-temperature values. The insignificancy of this anomaly is confirmed by the analysis of the DSC and thermogravimetry (Figures 3 and 4).

The DSC curve as a function of temperature shows that it is more intense than that of pure aluminium. At the temperature of 600°C, its intensity is four times greater. The shape of the two curves is superimposed. This behaviour is repeated in the thermogravimetry study, where TG intensity of the nanocomposite has doubled compared to that of aluminium.

**Figure 5:**
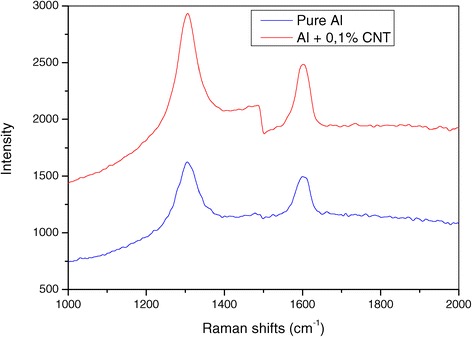
**Raman spectrum of nanocomposite Al + 0.1% CNT and pure aluminium.**

**Figure 6:**
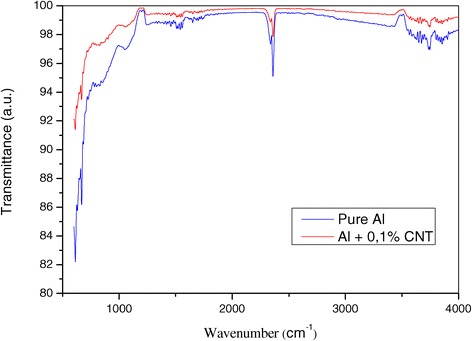
**Infrared spectrum of nanocomposite Al + 0.1% CNT and pure aluminium.**

The Raman spectrum of Al + 0.1% CNT nanocomposite (Figure 5) shows that G and D bonds are increased in their intensity and have shifted towards high frequencies, and the ratio *I*_D_/*I*_G_ [14,15] has also increased compared to that of pure aluminium prepared under the same conditions. As to the infrared diagram (Figure 6), the two curves are practically identical, except for intensity of the peaks of the nanomaterial and the pure material. Measurements of the tensile strength of the studied materials show a considerable increment in comparison with pure aluminium. Thus, the tensile strength of aluminium without nanotubes, produced by the same method as used in this study, is not greater than *σ*_B_ = 140 ± 10 MPa, while the same tensile strength of Al ribbon containing nanotubes is *σ*_B_ = 200 ± 10 MPa. This increment is caused by formation of clusters of nanotubes in the aluminium matrix, which raise the density of dislocations and block their mobility. This proves the importance and improved mechanical properties of nanocomposites containing carbon nanotubes [16]. Moreover, the obtained result is in agreement with those by the author researchers [17-21].

**Figure 7:**
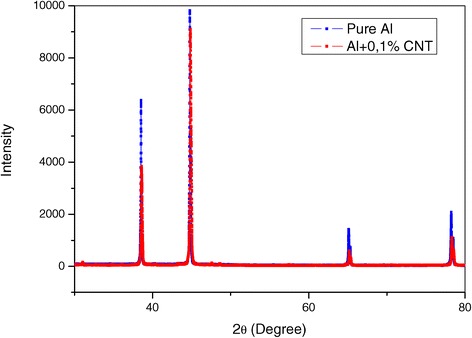
**X-ray diffraction pattern of nanocomposite Al + 0.1% CNT and pure aluminium.**

Based on the analysis of the X-ray diffraction spectrum of the nanocomposite and that of pure aluminium, produced in the same conditions (Figure 7), it can be concluded that spectral line broadening takes place upon CNT addition, as well as an insignificant variation of Al lattice spacing and the texture reinforcement.

## Conclusions

Introduction of carbon nanotubes to the aluminium matrix causes a significant decrease in the thermal expansion coefficient α_z_(T) as a function of temperature along the direction perpendicular to the rolling plane of the nanomaterial ribbon. This result is novel. This behaviour of TEC suggests that the expansion of the nanocomposite is governed by presence of a ‘phase’ of nanotubes, whose thermal expansion coefficient is close to zero and causes CNT of NCM to decrease.

Regardless of the measurement direction, α_z_(T) value is smaller than α_x_(T) and α_y_(T) over the entire temperature range. α_x_(T) and α_y_(T) demonstrate roughly the same behaviour up to the temperature of 400°C. Above this temperature, α_x_(T) becomes greater than α_y_(T). All the three thermal expansion coefficients are lower than those of pure aluminium and alloys produced by quick solidification methods. The thermal expansion coefficient of the studied nanocomposite is anisotropic. This anisotropy becomes the most considerable at high temperatures.

The results produced by different methods are consistent with the dilatometric data. The tensile strength of the studied NCM is higher than the one of pure aluminium. The DSC and TG curves demonstrate a greater intensity than in the case of pure aluminium. The peak intensities observed for the infrared and Raman spectra have evolved compared to those of the matrix.
